# Design and analysis of a performance monitoring system for a seed metering device based on pulse width recognition

**DOI:** 10.1371/journal.pone.0261593

**Published:** 2021-12-22

**Authors:** Zhengyuan Liu, Junfang Xia, Mengjie Hu, Jun Du, Chengming Luo, Kan Zheng

**Affiliations:** College of Engineering, Huazhong Agricultural University, Wuhan, China; University of Glasgow, UNITED KINGDOM

## Abstract

To realize real-time and accurate performance monitoring of large- and medium-sized seed metering devices, a performance monitoring system was designed for seed metering devices based on LED visible photoelectric sensing technology and a pulse width recognition algorithm. Through an analysis of the of sensing component pointing characteristics and seed motion characteristics, the layout of the sensing components and critical photoelectric sensing system components was optimized. Single-grain seed metering devices were employed as monitoring objects, and the pulse width thresholds for Ekangmian-10 cotton seeds and Zhengdan-958 corn seeds were determined through pulse width threshold calibration experiments employed at different seed metering plate rotational speeds. According to the seeding quantity monitoring experiments, when the seed metering plate rotational speed ranged from 28.31~35.71 rev/min, the accuracy reached 98.41% for Ekangmian-10 cotton seeds. When the seed metering plate rotational speed ranged from 13.78~19.39 rev/min, the seeding quantity monitoring accuracy reached 98.19% for Zhengdan-958 corn seeds. Performance monitoring experiments revealed that the qualified seeding quantity monitoring accuracy of cotton precision seed metering devices, missed seeding quantity monitoring accuracy, and reseeding quantity monitoring accuracy could reach 98.75%, 94.06%, and 91.30%, respectively, within a seeding speed range of 8~9 km/h. This system meets the requirements of real-time performance monitoring of large- and medium-sized precision seed metering devices, which helps to improve the operational performance of seeding machines.

## Introduction

Seeding is a critical link in agricultural production, and improving the seeding quality is a meaningful way to increase crop yield. At present, precision seeding technology is mainly employed to improve the seeding quality of crops [1]. As a critical seeder component, the performance of the seed metering device is an essential factor in ensuring the seeding quality. Certain seeder characteristics prevent the operator from directly monitoring the quality of the seeding operation. If the machine experiences reseeding or missed seeding, this could lead to a poor seed spacing uniformity, a low emergence rate, and an adverse impact on the agricultural production income [2,3]. Therefore, realizing real-time performance monitoring of seed metering devices is of great significance to improving the seeders’ working quality, which can provide major research and technology development support, such as real-time adjustment of the seeding rate and replanting of missed seeds.

The seed metering device performance is determined based on the seed spacing within a row. To assess the seed spacing, the performance (such as the seeding quantity, qualified seeding quantity, missed seeding quantity, and reseeding quantity) of seed metering devices should be monitored in real time during the seeding process. Performance monitoring systems for seed metering devices have been developed, and the majority of these devices are based on piezoelectric, capacitance, machine vision, and photoelectric sensing methods. Table 1 summarizes the principles, advantages and shortcomings of the above sensing methods based on the relevant literature.

**Table 1:** The principles, advantages and shortcomings of the sensing methods.

References	Sensing methods	Principles	Advantages	Shortcomings
[4–7]	Piezoelectric	The piezoelectric method uses pressure-sensitive elements to transform the impact of a seed into an electric signal	High piezoelectric voltage constants, fast frequency response, and low cost	Some seeds were deflected after touching the pressure-sensitive element. The seed falling track was altered, these seeds could touch the element again, resulting in overcounting
[8–12]	Capacitance	The change in the equivalent dielectric constant as the seeds pass through the detection area of the capacitance sensor causes a change in the capacitance output value	Complex ambient light and dust have less effect on capacitive sensors The method could detect more than one seed at a time	Capacitance sensors are easily influenced by the seed water content, machine vibrations, temperature, and parasitic capacitance Seeds with smaller volumes do not generate detectable variation in the dielectric coefficient
[13–17]	Machine vision	The method uses high-resolution or high-speed cameras to capture an image of the falling seeds and to process these images	High accuracy and versatility Can detect seeds on the soil surface	Extremely high cost and a large postprocessing effort is required The method has harsh environmental requirements and is not suitable for field operation
[18–29]	Photoelectric	Often includes a transmitter and receiver; if the light beam emitted by the transmitter is interrupted by seeds, the receiver output changes	Short response time, simple system construction, high stability and low cost It does not affect the trajectory of the seeds	The photoelectric method cannot detect two or more overlapping seeds The sensing component structure has a large impact on the detection accuracy

**Fig 1:**
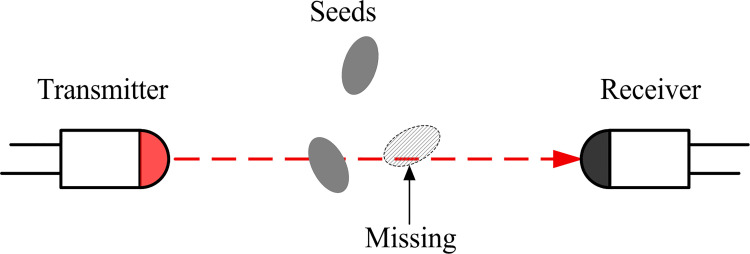
Schematic diagram of missing a seed during seed metering.

Detecting two or more seeds falling at the same time is a key issue in the development of seed metering device performance monitoring systems. Taking the photoelectric sensing method as an example, when two or more seeds overlapped between the transmitter and the receiver, only the seed closest to the transmitter could be detected, while the other seeds could be shaded and thus missed in the measurement, as shown in Fig 1. This was the main reason that the accuracy of the photoelectric sensor is unsatisfactory in monitoring the performance of the seed metering device, especially regarding the reseeding quantity.

Several studies have used different methods to improve the accuracy of seed metering performance monitoring systems. Wang et al. [4] developed a piezoelectric sensor for rice seeds using polyvinylidene fluoride (PVDF) material. Their results indicated that the detection accuracy declined with increasing advancing speed of the planter. Although the researchers specifically designed the seed guide tube to ensure that the rice seeds entering the guide tube would impact the sensor, the reseeding monitoring accuracy of the system was only 81.79%. Zhao et al. [5] designed an arc array seeding flow sensor based on piezoelectric ceramics to count the number of discharged seeds. The obtained results demonstrated that the maximum detection error of the sensor remained within 5% when the air pressure reached 166 Pa, and the seeding rate was lower than 170 seeds/s under the recommended operating parameters. Although this study optimized the design of the sensing unit layout and the overall structure of the sensor to effectively monitor the seeding quantity, the case of two or more seeds impacting the sensor at the same time was not discussed. In particular, there are no studies using piezoelectric methods that are capable of simultaneously monitoring two or more seeds falling at the same time. Zhou et al. [9] developed a capacitive sensor-based performance monitoring system for corn seeders. The system can obtain seeding performance information such as the seeding quantity, missed seeding quantity and reseeding quantity by calculating the capacitance pulse peak interval. The test results showed that the system had an accuracy of 94.6% for seeding quantity, 93.5% for missed seeding quantity and 88.1% for reseeding quantity. Later, the system was used for cotton seed metering device performance monitoring [10]. The test results showed that when the simulation velocity of the planter was 4.0 km/h, the system had an accuracy of 94.2% for seeding quantity, 92.3% for missed seeding quantity and 86.7% for reseeding quantity. A monitoring algorithm was designed in this study that can effectively distinguish between two seeds that fall together. However, the monitoring accuracy of this system is not high due to the high sensitivity of the capacitive sensor, which is easily disturbed [12].

Several studies have reported that machine vision and image processing methods achieved different degrees of success [13,14]. However, these methods suffer many disadvantages, such as the need for complex calibration and data processing operations, expensive cameras and software, high labor requirements, artificially lit environments, and long process times [15–17]. The machine vision method can effectively monitor two or more simultaneously falling seeds, but the method is extremely demanding in terms of the monitoring environment, thus limiting its application in agricultural production [11].

The photoelectric method is currently the most widely used method for monitoring the performance of seed metering devices because of its stable system and low cost [22]. Photoelectric sensors often employ diodes with 5 mm diameters as sensing components. Due to the large volumes of certain varieties of seeds, only a few light beams can monitor most falling seeds [23]. To avoid interference, there should be some clearance between any two adjacent light beams [22]. However, the trajectory of a falling seed cannot be controlled. Thus, falling seeds with smaller volumes can easily pass through these clearances, leading to missed detection [24]. In addition, the structural characteristics of the 5 mm diode will result in two seeds with smaller spacing (less than 5 mm) being detected as one seed. Certain photoelectric sensing systems with wider monitoring areas have been developed [26–29]. Al-Mallahi and Kataoka [27,28] built a multivariable model to estimate seed flow masses. Their model was based on a fiber sensor with a monitoring range that was wider than the diameter of the seed tube to cover the whole width of the area where the seeds fall without any clearance. Although the fiber sensor provided the opportunity to expand the detection width, it was still possible for two or more seeds to consecutively pass through the monitoring area without a space interval, which led to these seeds being counted only once. Wu et al. [29] used two fiber sensors, which were installed on the top and bottom of the monitoring area to detect two seeds that fell into the monitoring area at the same time. The system is designed to improve the accuracy of the reseeding quantity monitoring. However, two seeds crossing the first sensor at the same time may still cross the second sensor at the same time. In this case, the system will count the two seeds as one seed. As a result, the system still has a low precision of 87.71% for reseeding number monitoring. According to previous studies, although the monitoring width of the fiber sensor has been improved, the monitoring accuracy and robustness remain unacceptable. This might be caused by interference from machine vibrations, natural light, dust, etc. [27]. Moreover, in terms of the photoelectric sensing method, there is no seed-counting algorithm reported in the literature for counting two or more seeds falling at the same time.

The method we present in this paper differs from those reported in previous works. It is based on the photoelectric method to develop a performance monitoring system for seed metering devices based on a pulse width recognition algorithm suitable for large- and medium-sized seeds (such as cotton and corn seeds). This study not only retains many advantages of the photoelectric method, but can also effectively monitor two simultaneously falling seeds, filling a gap in earlier studies. The contributions of this study are as follows.

- Key components and circuits of the monitoring system were designed and implemented in hardware.
- An algorithm for monitoring two simultaneously falling seeds is proposed and conventional methods are introduced for comparison. The algorithm is implemented in software by writing a microcontroller program.
- The pulse width thresholds for Ekangmian-10 cotton seeds and Zhengdan-958 corn seeds were calibrated.
- A seed quantity metering monitoring accuracy comparison experiment was conducted between the proposed algorithm and an off-the-shelf method. The monitoring accuracy of this algorithm was confirmed.
- A performance monitoring experiment was performed on the selected cotton seed metering device. The monitoring accuracy for this system was compared to that described in previous articles to validate the effectiveness of this system.

## Hardware design

### Design of the key components

#### Diode positioning ring

**Fig 2:**
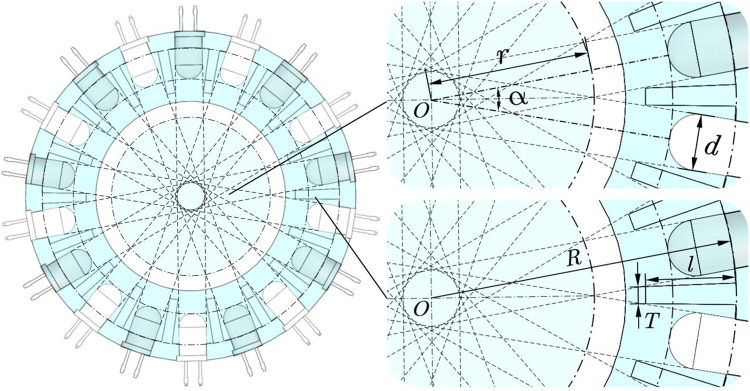
Structure diagram of the diode positioning ring.

A diode positioning ring was employed for the installation and positioning of high-brightness diodes and silicon photodiodes. It was equipped with diode positioning holes to ensure that the high-brightness diodes and silicon photodiodes were arranged in an equal-spacing circular array (Fig 2). The visible light beam emitted by the high-brightness diode was received by its radial corresponding silicon photodiode on the opposite side, and a pair of photoelectric detection units comprised the high-brightness diode and its radial opposite silicon photodiode. The circle tangent to the top of all the diodes was defined as the array circle. Under the joint action of several pairs of photoelectric detection units, the visible light beams emitted by the high-brightness diodes were stacked in the array circle and gathered at the center of the array circle to form a circular detection area concentric with the array circle without a blind area. According to the geometric relationship, the following can be obtained:

$$\{\begin{array}{l} \alpha =\frac{\pi }{x}\\ \sin\frac{\alpha }{2}=\frac{d}{2r} \end{array}$$ (1)

where *α* is the angle between adjacent diodes, rad; *x* is the number of photoelectric detection units, pairs; *d* is the diode diameter, mm; and *r* is the radius of the circular detection area without a blind area, mm.

According to Eq (1), the relationship between the number of photoelectric detection units and the radius of the circular detection area without a blind area can be obtained as follows:

$$x=\frac{\pi }{2\arcsin\frac{d}{2r}}$$ (2)

Based on the triaxial size of large- and medium-sized seeds (generally 3~12 mm), a transparent acrylic hard tube with an inner diameter of 26 mm and an outer diameter of 30 mm was adopted according to the light transmittance requirements of the photoelectric detection unit. The high-brightness diode employed was a 504 WC2Z-W2-3PF diode (Luckylight Electronics Co., LTD., Shenzhen, China) with a round head and white light, a 5-mm diameter, and a 60° luminous angle. The silicon photodiode was a 504PDC2B-3A device (Luckylight Electronics Co., LTD., Shenzhen, China) with a round head; it transparent, with a diameter of 5 mm and a response time of 45 *μs*. To ensure that there was no blind area in the seed guide tube, the radius of the circular nonblind area was *r* = 13 mm. Because the diameters of both the high-brightness diodes and silicon photodiodes were 5 mm, the diameter of the diodes *d* was 5 mm. According to Eq (2), *x* = 8.12 pairs could be calculated, and the value of *x* was rounded upward to the nearest integer to ensure that there was no blind area in the detection area. Finally, nine pairs of photoelectric detection units were applied to form a circular nonblind area with a radius of 14.18 mm.

**Fig 3:**
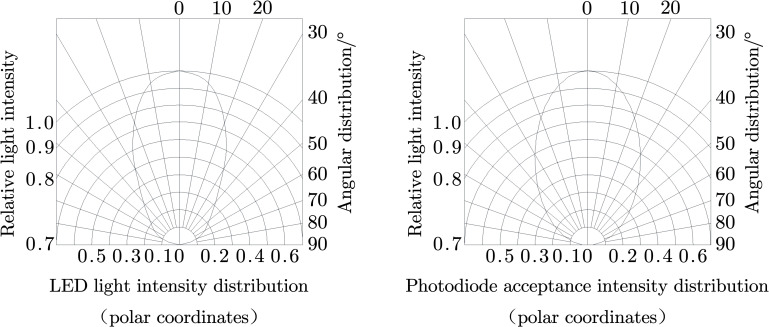
Relative intensity distribution of the diode.

An angle occurred between the diodes, and the spacing between adjacent diodes was small. Due to the pointing characteristics of the high-brightness diodes and silicon photodiodes [30,31] (Fig 3), the silicon photodiodes in the photoelectric detection unit could experience interference from the high-brightness diodes in the other photoelectric detection units. For the convenience of description, this phenomenon was defined as the interference between diodes. When a smaller seed passes through the detection area, due to the interference between diodes, the variation in the luminous flux received on the surface of the silicon photodiode is limited so that the voltage signal generated by the diode driving module is very weak. If the signal conditioning module amplifies the voltage signal but fails to reach the threshold voltage, missed detection occurs.

An anti-interference grid plate was added to the diode positioning ring cavity to reduce the interference between diodes and improve the monitoring accuracy. Each anti-interference grid plate was located at the bisector of the angle between two adjacent diodes. According to the geometric relationship, the following can be obtained:

$$l=R-r-\frac{T}{2\tan\frac{\alpha }{2}}$$ (3)

where *l* is the length of the anti-interference grid plate, mm; *R* is the inner circle radius of the diode positioning ring cavity, mm; and *T* is the thickness of the anti-interference grid plate, mm.

According to the structure and material transmittance of the LED photoelectric sensing system, the optimal value of the inner circle radius of the diode positioning ring cavity *R* was set to 27 mm, and the thickness of the anti-interference grid plate *T* was set to 1.5 mm. Therefore, *l* = 8.57 mm was calculated by Eq (3). Considering the machining accuracy of the parts and ensuring the integrity of the circular detection area with no blind area, the length of the anti-interference grid plate was set to 8.5 mm.

#### Beam plane conversion tube

**Fig 4:**
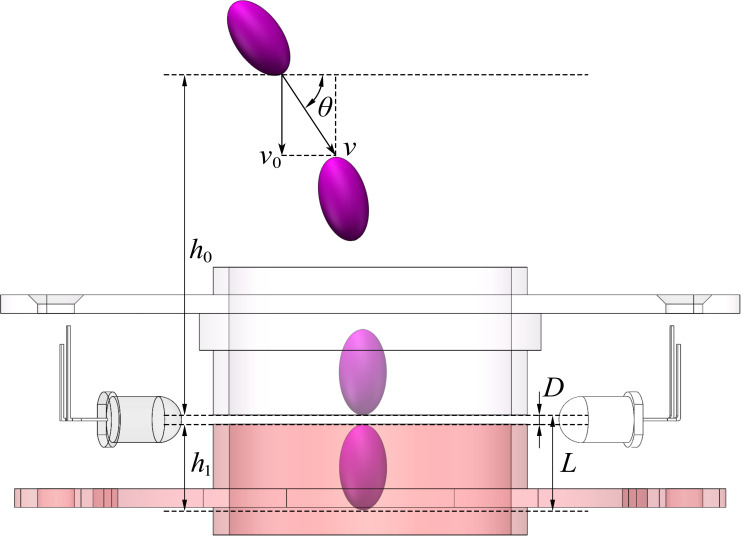
Schematic diagram of seeds passing through the detection area.

The beam plane conversion tube comprised upper and lower parts. There was a gap allowing light passage between the upper and lower tubes. The upper tube was integrated into the upper cover of the LED photoelectric sensing system, and the lower tube was an independent part (Fig 4). When a seed was discharged from the seed metering device and fell into the seed guide tube, according to Newton’s law of motion, we have the following:

$$\{\begin{array}{l} v=R_{0}\omega \\ v_{0}=v\sin\theta \\ v_{t}=v_{0}+gt\\ h_{0}=v_{0}t+\frac{1}{2}gt^{2} \end{array}$$ (4)

where *v* is the initial seed velocity, m/s; *R*_0_ is the seed metering plate radius, mm; *ω* is the angular velocity of the seed metering plate, rad/s; *v*_0_ is the vertical component of the initial seed velocity, m/s; *θ* is the angle between the initial seed velocity and horizontal plane, °; *v*_t_ is the velocity of the seed when it reaches the upper boundary of the detection area, m/s; *g* is the gravity acceleration, m/s^2^; *t* is the time it takes for the seed to travel from the drop point to the upper boundary of the detection area, s; and *h*_0_ is the vertical spacing from the seed drop point to the upper boundary of the detection area, mm.

The total spacing of the detection area through which the seed passes is as follows:

$$L=D+h_{1}=v_{t}t_{0}+\frac{1}{2}gt_{0}{}^{2}$$ (5)

According to Eq (5):

$$t_{0}=\frac{-v_{t}+\sqrt{v_{t}{}^{2}+2gL}}{g}$$ (6)

where *L* is the total spacing of the detection area through which the seed passes, mm; *D* is the thickness of the detection area, mm; *h*_1_ is the seed length, mm; and *t*_0_ is the time required for seeds to pass through the detection area, s.

According to Eqs (4)–(6), the time required for a seed to pass through the detection area is as follows:

$$t_{0}=\frac{\sqrt{{\left(R\omega \sin\theta \right)}^{2}+2g(h_{0}+l)}}{g}-\frac{\sqrt{{\left(R\omega \sin\theta \right)}^{2}+2gh_{0}}}{g}$$ (7)

The radius of the seed metering plate *R*_0_ was determined by the structure of the seed metering device. The angle between the initial seed velocity and horizontal plane *θ* was determined by the seed metering device installation mode. The vertical spacing from the seed drop point to the upper boundary of the detection area *h*_0_ could be determined according to the installation position of the LED photoelectric sensing system. The seed length *h*_1_ could be determined based on the seed type. According to Eq (7), when the seed metering device is operated at a constant speed *ω*, the smaller the thickness of the detection area *D*, the shorter the time required for seeds to pass through the detection area.

When the seed metering device performs the seed metering operation, due to the seed metering device discharging multiple seeds simultaneously, seeds colliding and impacting the seed guide tube and other reasons, the situation of one seed not having passed the detection area entirely when the next seed enters the detection area could occur when the spacing interval between seeds is very small, which could cause two seeds to pass through the detection area simultaneously. Consequently, the LED photoelectric sensing system outputs only one square pulse wave. The beam plane conversion tube could block part of the light beam and convert the stacked light beam emitted by the high-brightness diode into a thin plane of light. By changing the thickness of the gap that allows light to pass through the upper and lower tubes, the thickness of the detection area was compressed to shorten the time required for seeds to pass through the detection area to solve the missing detection problem attributed to the small spacing interval between two seeds. Considering the machining and assembly accuracy of the parts, the light gap thickness of the beam plane conversion tube was set to 0.5 mm. The thickness of the 5-mm detection area determined by the diode diameter was compressed to 0.5 mm, which further improved the detection accuracy.

### System circuit design

#### LED photoelectric sensing system circuit

**Fig 5:**
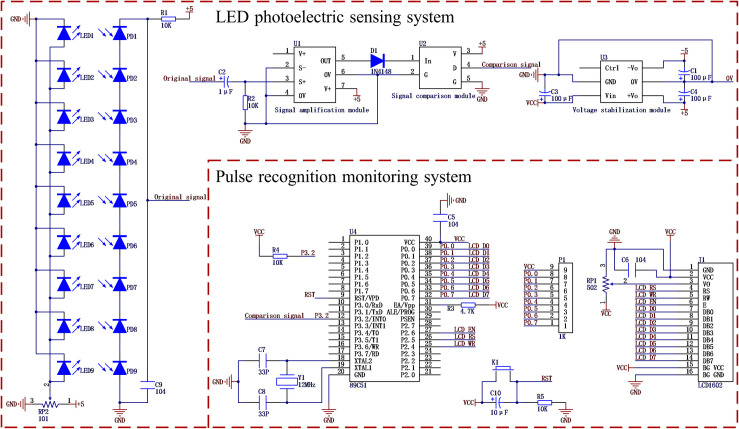
Circuit schematic diagram of the performance monitoring system for the seed metering device.

The circuit of the LED photoelectric sensing system consisted of three modules, i.e., a voltage stabilization module, a diode driving module, and a signal conditioning module (Fig 5). The power supply of the LED photoelectric sensing system originated from the 5-V lithium battery of the pulse recognition monitoring system. Because a voltage drop of approximately 0.3 V occurs upon three-core wire transmission, to ensure the stability of the LED photoelectric sensing system circuit, the voltage stabilization module was designed based on a VRA0505YMD-10 WR3 device (MORNSUN Technology Co., LTD., Guangzhou, China) to boost the input voltage of the LED photoelectric sensing system to 5 V and maintain its stability. The voltage stabilization module powered both the diode driving module and the signal conditioning module.

The diode driving module connected nine high-brightness diodes in parallel. Type 101 (100 Ω) precision adjustable resistors were adopted as current limiting resistors to ensure that the high-brightness diodes operated at the rated current level. Nine silicon photodiodes in series functioned under the action of reverse voltage. A resistance of 10 kΩ was generated by the current limiting resistor to prevent silicon photodiodes from being damaged due to high currents. A type 104 (0.1 μF) capacitor was applied as a filter capacitor to filter out a portion of the power supply noise and its AC components. The diode driving module converted the change in the luminous flux received on the surface of the silicon photodiode into a voltage change. This voltage change was output as the original signal. The original signal output port was connected with the signal input port S+ of the signal amplification module.

The signal conditioning module was designed based on an AD623AN instrumentation amplifier (Junteng Electronic Technology Co., LTD., Xian, China), 1N4148 diode (Zhongbao Electronics Co., LTD., Nanjing, China), and LM393 voltage comparator (Shidaihuaxin Technology Co., LTD., Shenzhen, China). The millivolt-level original signal output by the diode driving module was amplified to a maximum output voltage of 5 V by the AD623AN amplifier. The signal output port OUT of the signal amplification module was connected to the positive terminal of the 1N4148 diode. The negative terminal of the 1N4148 diode was connected to the signal input port In of the signal comparison module. The 1N4148 diode isolated the amplified signal via unidirectional conduction, which indicates that the forward voltage was activated, and the reverse voltage was cut off, leading to a voltage drop of approximately 0.7 V and a peak voltage signal value of approximately 4.3. The isolated signal was input to the LM393 voltage comparator, which was compared to the comparator threshold voltage. The comparator converted any analog signal greater than the threshold voltage into a square pulse wave signal. The signal output port D of the signal comparison module was connected to the P3.2/INT0 port of the microcontroller (STC89C516RD+). According to the working principle of the comparator, considering the interference impact of complex field vibration, ambient light, and dust on the voltage signal, the threshold voltage of the comparator was set to 2 V.

#### Pulse recognition monitoring system circuit

The pulse recognition monitoring system circuit included a lithium battery, a single-chip microcomputer minimum system, and an LCD module (Fig 5). The lithium battery was manufactured by Shenzhen Zhongshun New Energy Co., Ltd. LP505575X2S and had the following characteristics: voltage drop: 7.4 V; output: 5 V; rated current: 3 A; and capacity: 5000 mAh. The lithium battery supplied power to the LED photoelectric sensing system, single-chip microcomputer minimum system module, and LCD module and could meet the long-term work requirements of the monitoring system in the field.

The single-chip microcomputer minimum system was designed based on STC89C516RD+ (Hongjing Microelectronics Technology Co., LTD., Hefei, China). A 12-MHz crystal oscillator was selected as the external crystal oscillator of the single-chip microcomputer to facilitate the calculation of the delay time and ensure the accuracy of the timer. The reset circuit was designed by the resistance and capacitance. The output port of the square pulse wave signal of the LED photoelectric sensing system was connected to the external interrupt 0 port of the single-chip microcomputer via a three-core wire. The external interrupt 0 port was connected with a 10-kΩ pull-up resistor to improve the driving ability of the I/O port. The 8-bit parallel data port of the LCD module was connected to the P0 port of the single-chip microcomputer. Because the P0 port of the STC89C51 series single-chip microcomputer was an open-drain output, the external 1-kΩ resistance was implemented as the P0 port pull-up resistance to enable it to output a voltage level of 5 V (high level). The LCD module was designed based on the LCD1602 display (Youxin Electronic Technology Co., LTD., Shenzhen, China). A type 502 (5 kΩ) precision adjustable resistor was employed to adjust the contrast of the LCD display to ensure that the performance parameters of the seed metering device were accurately and clearly displayed. The lithium battery switch is used to control the power to this monitoring system.

### Hardware implementation

**Fig 6:**
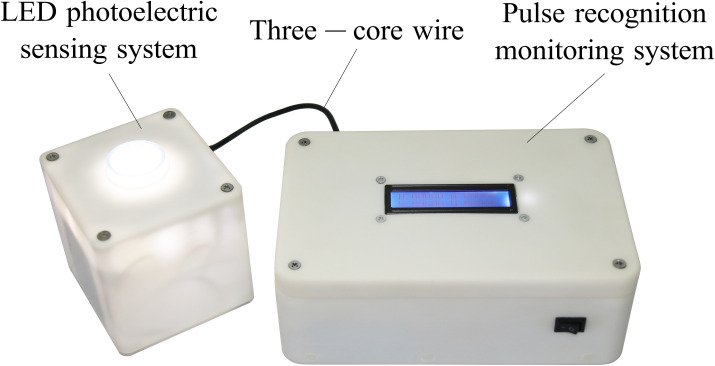
Picture of the monitoring system.

**Fig 7:**
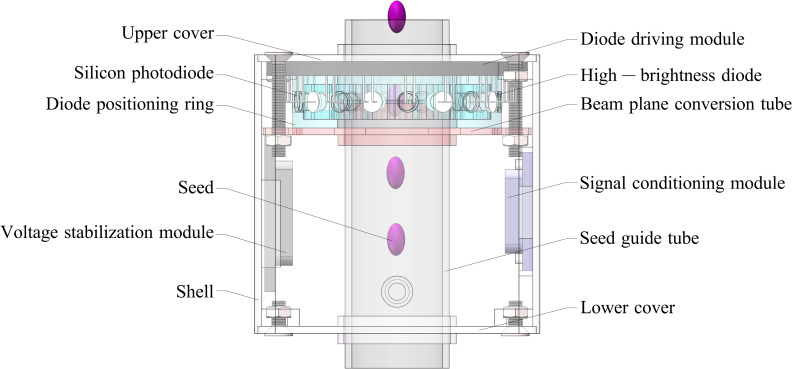
Structure of the LED photoelectric sensing system.

The monitoring system comprised an LED photoelectric sensing system and a pulse recognition monitoring system (Fig 6). The LED photoelectric sensing system mainly included a seed tube, diode positioning ring, beam plane conversion tube, silicon photodiode, high-brightness diode, diode drive module, voltage stabilization module, and signal conditioning module (Fig 7). The pulse recognition monitoring system consisted of an LCD screen, a single-chip microcomputer minimum system, and a lithium battery.

Under the joint action of the upper cover of the LED photoelectric sensing system and the beam plane conversion tube, the stacked light beam produced by the high-brightness diode is converted into a thin plane of visible light. During the seed metering operation, a seed falls into the LED photoelectric sensing system’s seed guide tube and passes through the thin visible light plane. Because the blocking effect of the seed on the visible light plane alters the visible light flux received by the silicon photodiode, the electric current in the circuit changes. Then, the diode driving module generated a voltage signal. The signal conditioning module amplified, shaped, and compared the voltage signal to the threshold voltage to form a square pulse wave. The square pulse wave is then sent to the pulse recognition monitoring system through the three-core wire and is connected to the external interrupt I/O port of the single-chip microcomputer as the external interruption source. When external interruption is triggered, the single-chip microcomputer calculates the width of a single pulse and the time interval between two pulses. The number of seeds (one or two) passing through the thin visible light plane is calculated by the pulse width and the seeding quantity is obtained. The qualified seeding, reseeding, and missed seeding quantities are determined by the time interval between pulses. The above performance parameters are monitored in real time on an LCD screen.

## Software design

### Seed metering monitoring quantity algorithm

Regarding seeding quantity monitoring, the main traditional monitoring method is the pulse counting method (PCM), which monitors the number of seeds by measuring the number of pulses. When a square pulse wave is output by the LED photoelectric sensing system, the seeding quantity of the pulse recognition monitoring system is increased by 1. However, when there is no spacing interval between two or more seeds, the LED photoelectric sensing system can only output one square pulse wave, and the PCM may misjudge this occurrence as one seed. Through observing the square pulse wave generated by a seed passing through the detection area with a DPO2014B oscilloscope (Tektronix Technology Co., LTD., Portland, America), it can be found that the square pulse wave width produced by a single seed falling differs from that produced by two or more seeds falling without a spacing interval. Based on the above phenomena, in this study, a pulse width recognition method (PRM) that monitors the number of seeds by measuring the pulse width is proposed. When a square pulse wave is output by the LED photoelectric sensing system, the pulse width is accurately measured by the pulse recognition monitoring system. Moreover, the PRM can determine whether a seed passing through the detection area is a single seed or two seeds with no spacing interval. Because the monitoring object of this system is a single-grain seed metering device, it is almost impossible for three or more seeds without spacing intervals to pass through the detection area, so this study does not discuss this situation.

**Fig 8:**
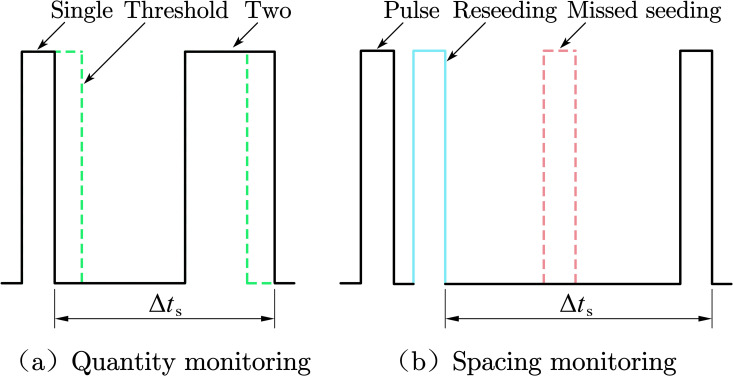
Schematic diagram of the seed dropping pulse.

Based on the PRM, the pulse width threshold concept was proposed. When the pulse width was smaller than or equal to the pulse width threshold, the seeding quantity of the pulse recognition monitoring system was increased by 1. When the pulse width was larger than the pulse width threshold, the seeding quantity of the pulse recognition monitoring system was increased by 2 (Fig 8(a)). Because the square pulse wave width is proportional to the time required for a seed to pass through the detection area, the square pulse waves generated by falling seeds were sampled at different rotational speed levels of the seed metering device. The square pulse wave width data were recorded and analyzed to determine the pulse width threshold at the different rotational speed levels.

### Seed spacing monitoring algorithm

According to the performance evaluation index of precision seeders in NY/T 1143–2006, which is the technical specification for the quality evaluation of drills, and the actual seeding operation requirements with regard to the seed spacing and advancing speed, we have the following:

$$\Delta t=\frac{\bar{d}}{V}$$ (8)

where Δ*t* is the theoretical time interval between two adjacent seeds, s; $\bar{d}$ is the theoretical seed spacing, m; and *V* is the advancing speed of the precision seeder, m/s.

According to GB/T 6973–2005, which is a testing method for single-seed drills (precision drills), if the actual seed spacing was smaller than or equal to 0.5 times the theoretical seed spacing, reseeding occurred. If the actual seed spacing was larger than 1.5 times the theoretical seed spacing, missed seeding ensued. Furthermore, if the actual seed spacing varied between these two values, qualified seeding occurred.


$$\{\begin{array}{l} d\leq 0.5\bar{d}\;(\mathrm{Reseeding})\\ d>1.5\bar{d}\;(\mathrm{Missed}\;\mathrm{seeding})\\ 0.5\bar{d}<d\leq 1.5\bar{d}\;(\mathrm{Qualified}\;\mathrm{seeding}) \end{array}$$ (9)

Here, *d* is the actual seed spacing, m. According to Eqs (8) and (9) can be rewritten as follows:

$$\{\begin{array}{l} d\leq 0.5\Delta tV\;(\mathrm{Reseeding})\\ d>1.5\Delta tV\;(\mathrm{Missed}\;\mathrm{seeding})\\ 0.5\Delta tV<d\leq 1.5\Delta tV\;(\mathrm{Qualified}\;\mathrm{seeding}) \end{array}$$ (9)

The actual seed spacing of the precision seeder under different working conditions is as follows:

$$d=\Delta t_{s}V$$ (10)

where Δ*t*_s_ is the actual time interval between two adjacent seeds, s. According to Eqs (10) and (11), we have the following:

$$\{\begin{array}{l} \Delta t_{s}\leq 0.5\Delta t\;(\mathrm{Reseeding})\\ \Delta t_{s}>1.5\Delta t\;(\mathrm{Missed}\;\mathrm{seeding})\\ 0.5\Delta t<\Delta t_{s}\leq 1.5\Delta t\;(\mathrm{Qualified}\;\mathrm{seeding}) \end{array}$$ (11)

Therefore, the monitoring system could convert the actual seed spacing *d* into the actual time interval Δ*t*_s_ between two adjacent seeds, as shown in Fig 8(b). Thus, a monitoring algorithm for seed spacing reseeding and missed seeding determination for the precision seeder could be established. The single-chip microcomputer calculated the actual time interval Δ*t*_s_ between two adjacent seeds by measuring the falling edge of two adjacent square pulse waves. Comparing Δ*t*_s_ to the theoretical time interval Δ*t* between two adjacent seeds under various working conditions, if Δ*t*_s_ ≤ 0.5Δ*t*, this was categorized as reseeding, but if Δ*t*_s_ > 1.5Δ*t*, this was categorized as missed seeding. Moreover, if 0.5Δ*t* < Δ*t*_s_ ≤ 1.5Δ*t*, the seed spacing was suitable.

## Software implementation

### Program based on the PRM

**Fig 9:**
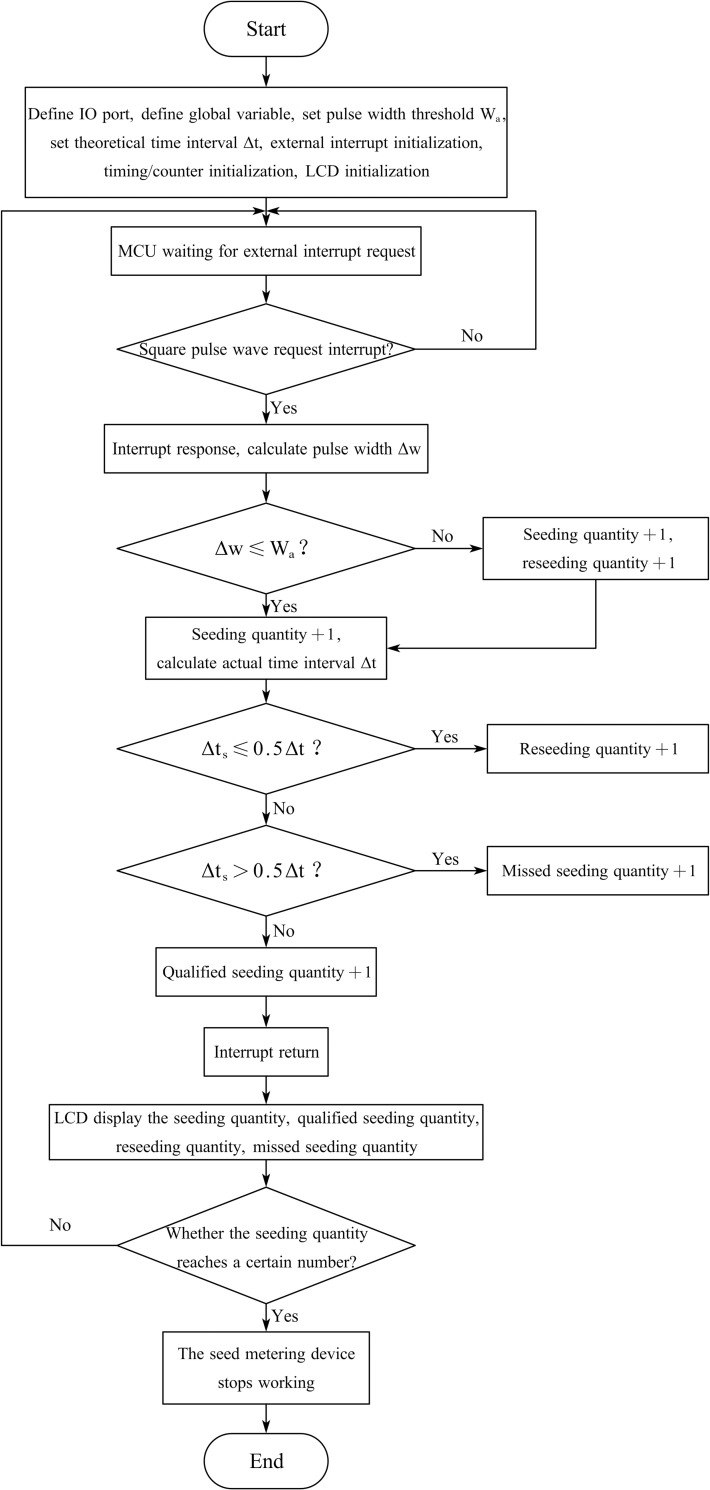
Flow chart of the program based on the PRM.

Based on the PRM and C language, a single-chip microcomputer program was written in Keil uvisin4 (V4.02) software, which was used to control the pulse recognition monitoring system to receive the square pulse wave signal stemming from the LED photoelectric sensing system, identify the pulse width, assess the adjacent pulse time interval, and finally display the performance parameters of the seed metering device. A program flow chart of the single-chip microcomputer is shown in Fig 9. After the single-chip microcomputer was activated, the program was initialized, and relevant IO ports and variables were defined, including setting the pulse width threshold and the theoretical time interval between adjacent seeds and initializing the external interrupt 0 port, timing/counter 0 port, timing/counter 1 port, and LCD1602 display.

After initialization, the single-chip microcomputer entered the external interrupt request state. When the square pulse wave was applied as the interrupt source to request external interruption, the single-chip microcomputer responded to this interruption. First, the microcontroller calculated the pulse width and compared this to the pulse width threshold to determine the number of seeds (1 or 2). Then, the actual time interval between adjacent pulses was calculated and compared to the theoretical time interval. Moreover, the program judged whether the actual seed spacing was suitable, and the seeding quantity, qualified seeding quantity, reseeding quantity, and missed seeding quantity were finally displayed on the LCD1602 display. When the seeding quantity reaches a certain number, the seed metering device stops working.

### Program based on the PCM

**Fig 10:**
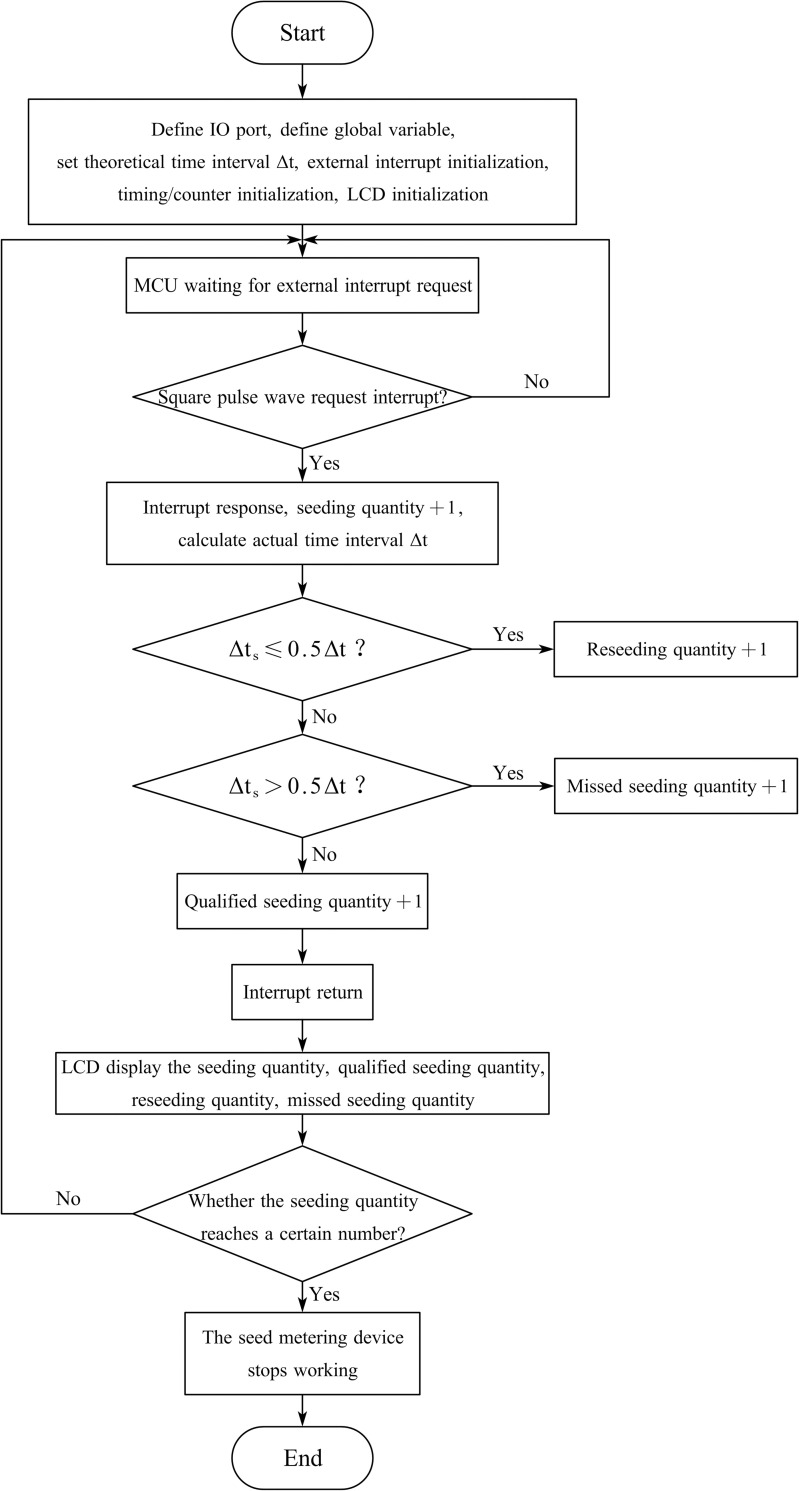
Flow chart of the program based on the PCM.

A single-chip microcomputer program based on the PCM was written as a comparative monitoring program in the same development environment as that of the PRM. A program flow chart is shown in Fig 10. After program initialization was completed, the single-chip microcomputer entered the external interrupt request state. When the square pulse wave was employed as the interrupt source to request an external interrupt, the single-chip microcomputer responded and increased the seeding quantity by 1. The actual time interval between adjacent pulses was calculated and compared to the theoretical time interval to determine whether the actual seed spacing was suitable. Finally, the seeding quantity, qualified seeding quantity, reseeding quantity, and missed seeding quantity were displayed on the LCD1602 display. When the seeding quantity reaches a certain number, the seed metering device stops working.

## System tests and results

**Fig 11:**
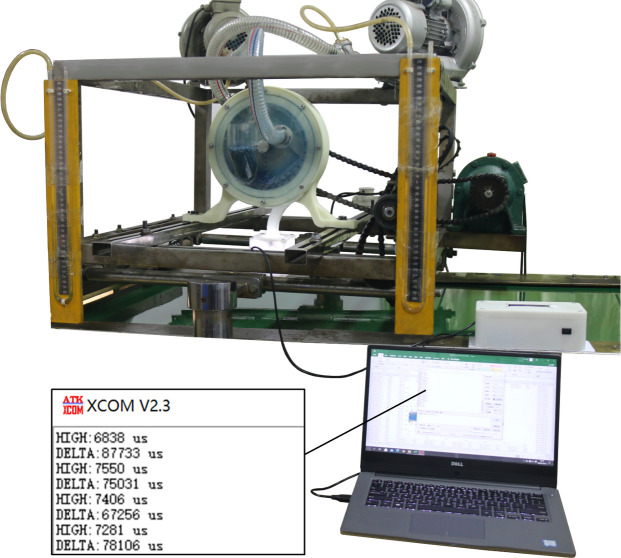
Bench test device.

The test was carried out at the engineering base of Huazhong Agricultural University. The test equipment mainly included a JPS-12 machine vision precision seed metering device performance monitoring test bed, cotton precision seed metering device, corn and soybean dual-purpose precision seed metering device, seed box, upper computer, and a large- and medium-sized seed metering device performance monitoring system. The data collection software was XCOM (V2.3, Xingyi Electronic Co., Ltd., Guangzhou, China). The experimental materials included Ekangmian-10 cotton seeds and Zhengdan-958 corn seeds. The test device is shown in Fig 11.

### Pulse width threshold calibration experiment

According to Eq (7), the rotational speed of the seed metering plate of the seed metering device is inversely proportional to the time required for a seed to pass through the detection area. Ekangmian-10 cotton seeds and Zhengdan-958 corn seeds are irregular, and the triaxial sizes are quite different. The square pulse wave width was measured at different seed metering plate rotational speeds to research the relationship between the rotational speed of the seed metering plate and the square pulse wave width output by the LED photoelectric sensing system. Furthermore, the pulse width threshold was calibrated.

**Fig 12:**
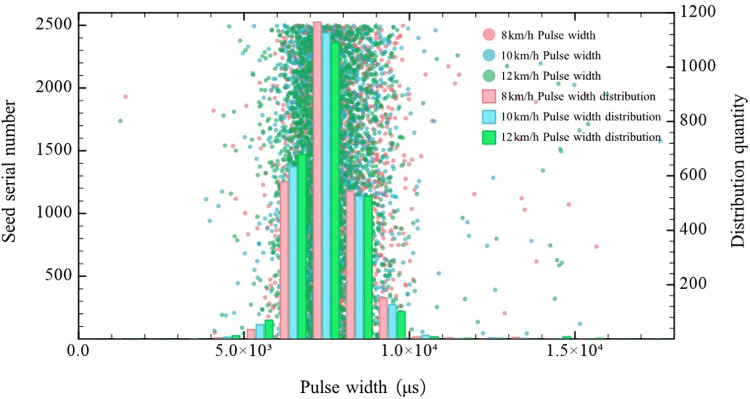
Pulse width distribution for the Ekangmian-10 cotton seeds.

Regarding the Ekangmian-10 cotton seeds, the seed plate rotational speed of the cotton precision seed metering device was set to 23.81, 29.76, and 35.71 rev/min (corresponding to advancing speeds of 8, 10, and 12 km/h, respectively). The square pulse wave width produced by the falling seeds was measured at three seed plate rotational speeds, and the upper computer recorded the square pulse wave width. Two thousand five hundred pulse widths were recorded at each rotational speed. Combined with the pulse width data, counting occurred in segments, and the pulse width distribution at the different rotational speeds were obtained, as shown in Fig 12.

According to Fig 12, at the three rotational speeds of 23.81, 29.76, and 35.71 rev/min, the pulse widths of the Ekangmian-10 cotton seeds mainly ranged from 5 to 10 ms. Consequently, the pulse width threshold for the Ekangmian-10 cotton seeds based on the PRM was set to 10 ms. When the pulse width generated by a cotton seed passing through the detection area was greater than 10 ms, the monitoring system registered this occurrence as two seeds, the seeding quantity was increased by 2, and the reseeding quantity was increased by 1. As a result, the problem whereby a small spacing interval between two adjacent seeds or partial overlap between two adjacent seeds was categorized as a single seed by the PCM could be solved.

**Fig 13:**
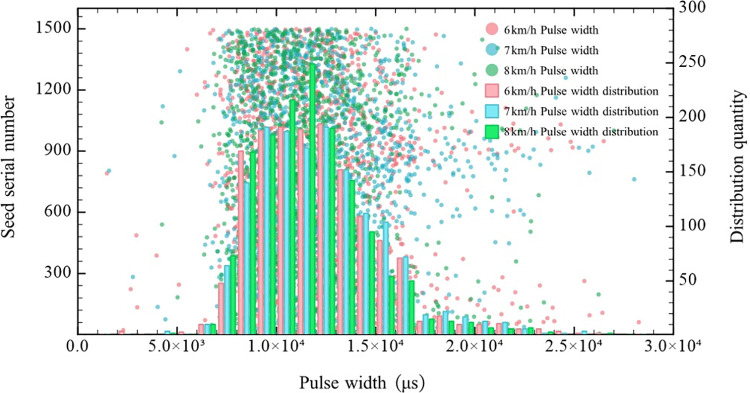
Pulse width distribution for the Zhengdan-958 corn seeds.

For the Zhengdan-958 corn seeds, the seed metering plate speed of the dual-purpose precision seed metering device for corn and soybean seeds was set to 13.78, 16.66 and 19.39 rev/min (corresponding to advancing speeds of 5, 6, and 7 km/h, respectively). The square pulse wave width produced by the falling seeds was measured at three seed metering plate speeds, and the upper computer recorded the square pulse wave width. One thousand five hundred pulse widths were recorded at each speed. Combined with the pulse width data, segments were counted, and the pulse width distribution at the different rotational speeds were obtained, as shown in Fig 13.

According to Fig 13, the seed pulse width for the Zhengdan-958 corn seeds largely ranged from 7 to 17 ms at the three rotational speeds of 13.78, 16.66, and 19.39 rev/min. Therefore, the seed pulse width threshold for the Zhengdan-958 corn seeds monitored by the PRM was set to 17 ms.

### Seeding quantity monitoring experiment

The experiment was carried out at different seed metering plate rotational speeds and repeated three times. In each experiment, the PCM and PRM were implemented simultaneously to monitor the seeding quantity. When the seeding quantity reached a certain amount (approximately 1000 cotton seeds and approximately 500 corn seeds), the seed metering device stopped working and the actual seeding quantity was manually counted. Compared to the actual seeding quantity and the seeding quantity monitored by the two methods, the monitoring accuracy of the system regarding the seeding quantity was computed, as in Eq (12) The results are listed in Table 2.

$$A_{n}=\frac{\mathrm{Min}(N_{1},\;N_{2})}{\mathrm{Max}(N_{1},\;N_{2})}\times 100\%$$ (12)

where *A*_n_ is the monitoring accuracy of seeding quantity, %; *N*_1_ is the actual seeding quantity; and *N*_2_ is the seeding quantity monitored by this monitoring system.

**Table 2:** Results of seeding quantity monitoring.

Seed type	Seed metering plate rotational speed (r·min^-1^)	Actual value	PCM monitoring value	PCM monitoring accuracy (%)	PRM monitoring value	PRM monitoring accuracy (%)
Ekangmian-10	23.81	1000	978	97.80	996	99.60
		1002	981	97.90	1006	99.60
		1001	964	96.30	999	99.80
	29.76	1002	972	97.01	994	99.20
		1000	970	97.00	993	99.30
		1004	973	96.91	996	99.20
	35.71	1008	979	97.12	992	98.41
		1006	970	96.42	991	98.51
		1000	966	96.60	985	98.50
Zhengdan-958	13.78	501	490	97.80	503	99.60
		502	489	97.41	498	99.20
		506	498	98.42	510	99.21
	16.66	507	473	93.29	499	98.42
		494	477	96.56	486	98.38
		495	482	97.37	496	99.80
	19.39	497	477	95.98	488	98.19
		502	481	95.82	493	98.21
		501	483	96.41	492	98.20

According to Table 2, the monitoring accuracy of PRM monitoring in each experiment was greater than that of PCM monitoring. Regarding the Ekangmian-10 cotton seeds, the monitoring accuracies of the PCM and PRM were not lower than 96.30% and 98.41%, respectively. For the Zhengdan-958 corn seeds, the relative errors of PCM and PRM monitoring were not lower than 93.92% and 98.19%, respectively.

### Performance monitoring experiment of the seed metering device

In the experiment, a cotton precision seed metering device was installed on the JPS-12 machine vision precision seed metering device performance monitoring test bed. Additonally, the LED photoelectric sensing system was installed at the end of the seed guide tube to realize synchronous monitoring of the seed metering device with the monitoring system and the JPS-12 test bed. In the experiment, three seeding advancing speeds of 8, 9, and 10 km/h (corresponding to rotational speeds of 23.81, 26.79 and 29.76 rev/min, respectively) were selected. Moreover, the LED photoelectric sensing system was placed close to the sticky belt. Then, the data for 1000 seeds were collected at each speed, and the data from the pulse recognition monitoring system were compared to that from the JPS-12 test bed. The monitoring accuracy of the seed metering performance metrics was computed, as in Eq (13). The results of the experiment are listed in Table 3.

$$A_{m}=\frac{\mathrm{Min}(M_{1},\;M_{2})}{\mathrm{Max}(M_{1},\;M_{2})}\times 100\%$$ (13)

where *A*_m_ is the monitoring accuracy of the seed metering performance metrics, %; *M*_1_ is the value of seed metering performance metrics monitored by the JPS-12 test bed; and *M*_2_ is the value of seed metering performance metrics monitored by this monitoring system, which corresponded to *M*_1_.

**Table 3:** Seed metering performance monitoring results.

Seeding speed (km·h^-1^)	Project	Qualified seeding quantity	Missed seeding quantity	Reseeding quantity
8	JPS-12 value	906	44	50
	monitoring system value	904	42	54
	accuracy (%)	99.78	95.45	92.59
9	JPS-12 value	830	101	69
	monitoring system value	842	95	63
	accuracy (%)	98.57	94.06	91.3
10	JPS-12 value	830	93	77
	monitoring system value	828	92	80
	accuracy (%)	99.76	98.92	96.25

Table 3 reveals that, compared to the JPS-12 test bed, the system-based qualified seeding quantity monitoring accuracy was not lower than 98.57%, i.e., a high monitoring accuracy was achieved. In contrast, the monitoring system monitoring accuracy values regarding the missed seeding and reseeding quantities were slightly lower. The missed seeding quantity monitoring accuracy was not lower than 94.06%, and that of the reseeding quantity was not lower than 91.30%.

## Discussion

The seeding quantity monitoring results showed that the PRM proposed in this study significantly improved the monitoring accuracy over the traditional PCM, especially for Zhengdan-958 corn seeds. The seed metering performance monitoring results were analyzed. The reason for the slightly lower missed seeding quantity monitoring accuracy was that a small number of seeds categorized as suitable by the monitoring system collided with the seed guide tube or were deflected by the sticky belt after passing through the detection area. This resulted in the seed spacing on the sticky belt of the test bed being 1.5 times greater than the theoretical seed spacing, which was registered as missed seeding by the JPS-12 test bed. The main reason for the slightly lower accuracy of reseeding quantity monitoring was that the working principle of the monitoring system, which involved the conversion of the actual seed spacing into the time interval between adjacent seeds through the monitoring optical signal, resulted in situations where two completely overlapping seeds or multiple seeds with continuous partial overlaps and complete overlaps when passing through the detection area generated a single pulse width that was smaller than or equal to the pulse width threshold set by the monitoring system. This type of occurrence was thus registered as one seed by the monitoring system.

The monitoring accuracy of the monitoring system based on pulse width recognition can be compared with those found in previous studies. Several researchers have developed monitoring systems to monitor both cotton and corn seeds [9,10] and can be considered for comparison with the proposed system. The seeding quantity monitoring accuracy results for cotton seeds and corn seeds in [9,10] were 94.6% and 94.2%, respectively; however, the accuracy results of this research were 98.41% and 98.19%, respectively. In [10], the missed seeding quantity monitoring accuracy and reseeding quantity monitoring accuracy of cotton seed metering devices were 92.3% and 86.7%, respectively; nevertheless, the accuracy results of this research were 94.06% and 91.30%, respectively. The proposed system outperforms the system reported in a previous study in terms of seeding quantity monitoring accuracy, missed quantity monitoring accuracy and reseeding quantity monitoring accuracy. In particular, the greatest improvement is achieved in the monitoring accuracy of reseeding quantity.

## Conclusions

(1) With the use of a high-brightness diode and silicon photodiode, a performance monitoring device for a seed metering device was designed based on the photoelectric method, and a prototype was produced and tested. To solve the problems of the low temporal resolution of traditional diode detection, the occurrence of a blind detection area and interference between diodes, a diode positioning ring and beam plane conversion tube were designed. An LED photoelectric sensing system circuit and pulse recognition monitoring system circuit were also developed.

(2) A pulse width recognition monitoring algorithm was established, and the traditional pulse counting monitoring method was introduced as a comparison. Based on the above two monitoring methods, a single-chip microcomputer program was compiled. The monitoring system was able to realize real-time monitoring and display of the seeding quantity, qualified seeding quantity, missed seeding quantity, and reseeding quantity and was able to transmit pulse width and pulse time interval data to the upper computer through a serial port.

(3) In the pulse width threshold calibration experiment, the pulse width thresholds for Ekangmian-10 cotton seeds and Zhengdan 958 corn seeds were determined, at 10 and 17 ms, respectively. The monitoring experiment of the seeding quantity indicated that the precision of the PRM was significantly higher than that of the traditional PCM. The seeding quantity monitoring accuracy for the Ekangmian-10 cotton seeds and that for the Zhengdan 958 corn seeds were 98.41% and 98.19%, respectively. The performance monitoring experiment of the seed metering device revealed that the qualified seeding quantity monitoring accuracy of the cotton precision seed metering devices, missed seeding quantity monitoring accuracy, and reseeding quantity monitoring accuracy could reach 98.75%, 94.06%, and 91.30%, respectively. The performance monitoring system could effectively monitor the performance parameters of the seed metering device and meet the real-time indoor performance monitoring requirements of the seed metering device.

## Supporting information

S1 Fig 6 original photo. (JPG)

S2 Fig 11 original photo. (JPG)

S3 Fig 12 data. (XLSX)

S4 Fig 14 data. (XLSX)
