# Supplementary figures and images for: Design and analysis of a performance monitoring system for a seed metering device based on pulse width recognition
Source: PLoS One. 2021 Dec 22;16(12):e0261593. doi: 10.1371/journal.pone.0261593 (PMC8694462; doi:10.1371/journal.pone.0261593)

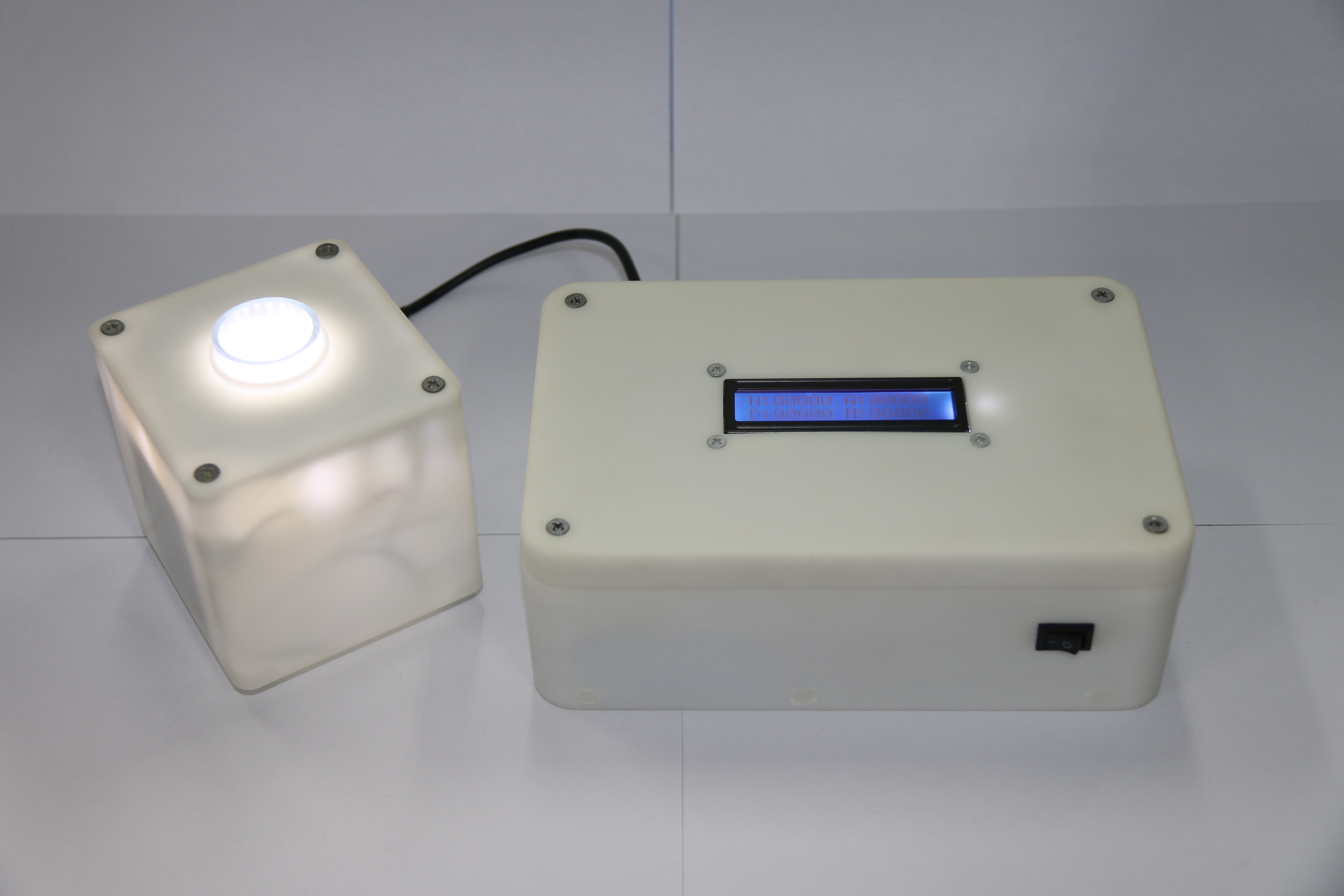

Supplement: S1 Fig — (JPG) [file pone.0261593.s001.jpg]

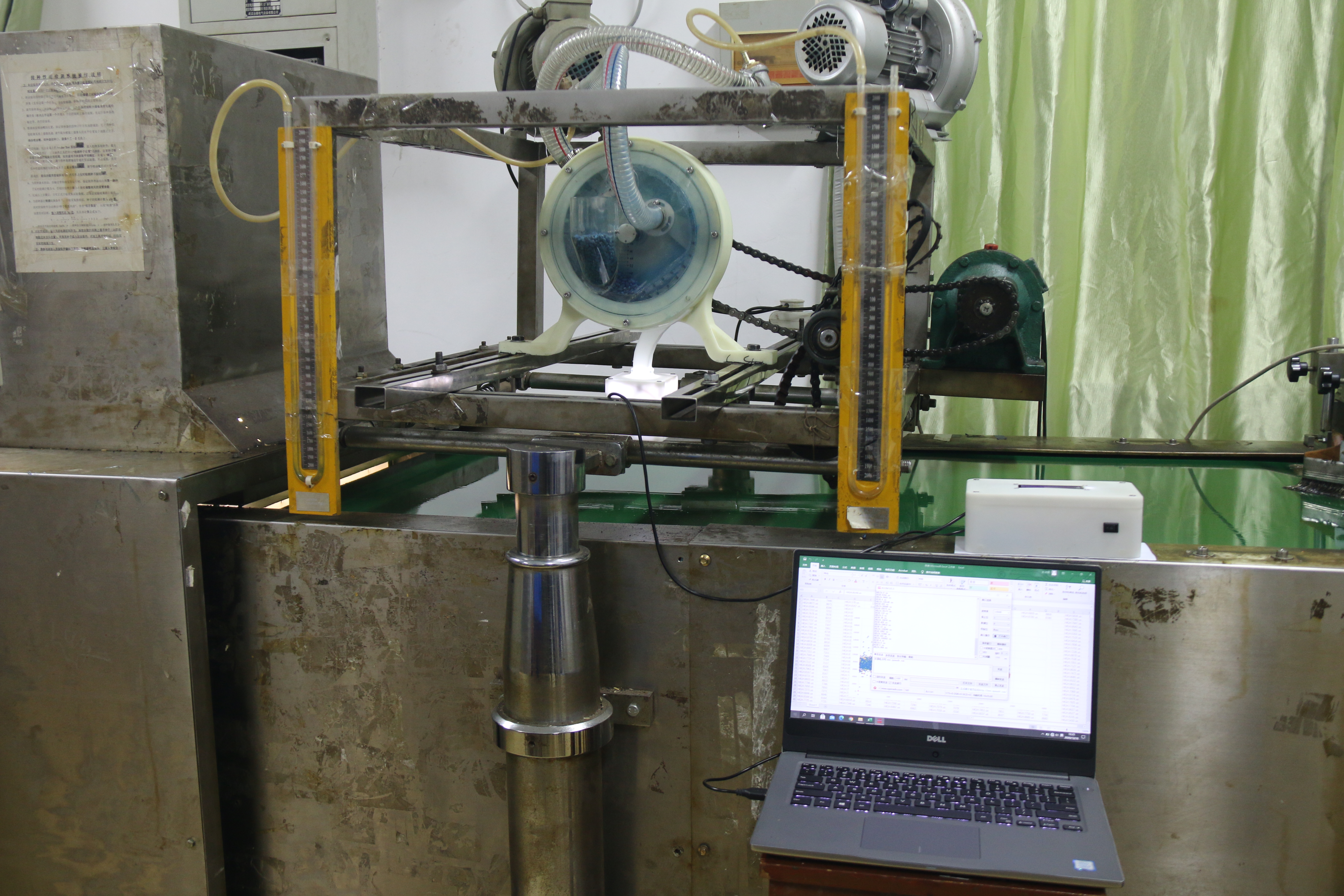

Supplement: S2 Fig — (JPG) [file pone.0261593.s002.jpg]
